# Continuous Glucose Monitor Metrics That Predict Neonatal Adiposity in Early and Later Pregnancy Are Higher in Obesity Despite Macronutrient-Controlled Eucaloric Diets

**DOI:** 10.3390/nu16203489

**Published:** 2024-10-15

**Authors:** Teri L. Hernandez, Sarah S. Farabi, Rachael E. Van Pelt, Nicole Hirsch, Emily Z. Dunn, Elizabeth A. Haugen, Melanie S. Reece, Jacob E. Friedman, Linda A. Barbour

**Affiliations:** 1 College of Nursing, University of Colorado, Anschutz Medical Campus, Aurora, CO 80045, USA; nicole.hirsch@cuanschutz.edu (N.H.); emily.2.dunn@cuanschutz.edu (E.Z.D.); 2 Division of Endocrinology, Metabolism, and Diabetes, Department of Medicine, University of Colorado School of Medicine, Aurora, CO 80045, USA; elizabeth.haugen@cuanschutz.edu; 3 Children’s Hospital Colorado, Anschutz Medical Campus, Aurora, CO 80045, USA; 4 Division of Nutritional Science & Obesity Medicine, Department of Medicine, Washington University, St. Louis, MO 63130, USA; ssfarabi@wustl.edu; 5 Goldfarb School of Nursing at Barnes-Jewish College, St. Louis, MO 63110, USA; 6 Division of Geriatric Medicine, Department of Medicine, University of Colorado School of Medicine, Aurora, CO 80045, USA; revanpelt@gmail.com; 7 Division of Neonatology, Department of Pediatrics, University of Colorado School of Medicine, Aurora, CO 80045, USA; melanie.reece@state.co.us; 8 Harold Hamm Diabetes Center, The University of Oklahoma Health Science Center, Oklahoma City, OK 73104, USA; jed-friedman@ouhsc.edu; 9 Division of Maternal-Fetal Medicine, Department of Obstetrics and Gynecology, University of Colorado School of Medicine, Anschutz Medical Campus, Aurora, CO 80045, USA; lynn.barbour@cuanschutz.edu

**Keywords:** pregnancy, obesity, diet, eucaloric, CGM, glucose metrics, infant adiposity

## Abstract

Background: Fasting glucose is higher in pregnancies with obesity (OB); less is known about postprandial (PP) and nocturnal patterns when the diet is eucaloric and fixed or about the continuous-glucose-monitor (CGM) metrics that predict neonatal adiposity (NB%fat). We hypothesized that continuous glucose monitors (CGMs) would reveal higher glycemia in OB vs. normal weight (NW) during *Early* (14–16 weeks) and *Later* (26–28 weeks) gestation despite macronutrient-controlled eucaloric diets and elucidate unique predictors of NB%fat. Methods: In a prospective, parallel-group comparative study, a eucaloric diet (NW: 25 kcal/kg; OB: 30 kcal/kg) was provided (50% carbohydrate [20% simple/30% complex; of total calories], 35% fat, 15% protein) to Early and Later gestation groups wearing a blinded CGM for three days. CGM metrics (mean fasting; 1 h and 2 h PP; daytime and nocturnal glucose; percent time-in-range (%TIR: 63–140 mg/dL); PP excursions; and area-under-the-curve [AUC]) were interrogated between groups and as predictors of NB%fat by dual X-ray absorptiometry(DXA). Results: Fifty-four women with NW (BMI: 23 kg/m^2^; *n* = 27) and OB (BMI: 32; *n* = 27) provided their informed consent to participate. Early, the daytime glucose was higher in OB vs. NW (mean ± SEM) (91 ± 2 vs. 85 ± 2 mg/dL, *p* = 0.017), driven by 2 h PP glucose (95 ± 2 vs. 88 ± 2, *p* = 0.004). Later, those with OB exhibited higher nocturnal (89 ± 2 vs. 81 ± 2), daytime (95 ± 2 vs. 87 ± 2), 1 h (109 ± 3 vs. 98 ± 2), and 2 h PP (101 ± 3 vs. 92 ± 2) glucose (all *p* < 0.05) but no difference in %TIR (95–99%). Postprandial peak excursions for all meals were markedly blunted in both the Early (9–19 mg/dL) and Later (15–26 mg/dL). In OB, the Later group’s 24 h AUC was correlated with NB%fat (r = 0.534, *p* = 0.02). Despite similar weight gain, infants of OB had higher birthweight (3528 ± 107 vs. 3258 ± 74 g, *p* = 0.037); differences in NB%fat did not reach statistical significance (11.0 vs. 8.9%; *p* > 0.05). Conclusions: Despite macronutrient-controlled eucaloric diets, pregnancies with OB had higher glycemia Early and Later in gestation; the Later 24 h glucose AUC correlated with NB%fat. However, glycemic patterns were strikingly lower than current management targets.

## 1. Introduction

The high prevalence of obesity in young women continues to challenge the landscape of pregnancy and obstetric care. In 2015, the American College of Obstetricians and Gynecologists recognized maternal obesity as the most common threat to health in women of reproductive age [1]; its impact on short-term and particularly long-term offspring health is acknowledged and requires further elucidation. Although typically associated with maternal diabetes, large-for-gestational-age (LGA) infants are most common in pregnancies affected by obesity outside of diabetes [2,3]. Even more predictive than LGA or birthweight (BW) of child obesity risk is infant body composition, particularly neonatal adiposity (%NB fat) [4]. Elevated pre-pregnancy BMI, excessive gestational weight gain, and exacerbated maternal insulin resistance associated with heightened exposure to excess glucose and lipids have been implicated in obesity-associated fetal macrosomia [5].

We previously reported normative data on patterns of glycemia in normal pregnancy by systematic review [6], and subsequently demonstrated using continuous glucose monitoring (CGM) that pregnant women with obesity (OB) had higher 24 h patterns of glycemia compared to normal-weight (NW) controls both ad libitum and when their diet was controlled, although analysis on each diet was limited to 24 h [7]. It was surprising that despite the higher patterns of glycemia in OB, the strongest predictor of NB%fat was fasting triglycerides (TG) at 14 weeks gestation, and fasting free fatty acids (FFA) at 28 weeks. In this new cohort, when the dietary macronutrient content was controlled and calories fixed according to maternal BMI during all metabolic testing, we previously reported that independent of glucose, 1 h and 2 h postprandial (PP) TGs at 14–16 weeks gestation were, in fact, the strongest predictors of NB%fat [8].

This study addresses the long-believed posit that women with OB have higher 24 h glycemia, especially apparent with CGM technology, and this may explain higher BW, LGA, and adiposity. However, whether differences in CGM metrics are due to the OB vs. NW metabolic phenotype or differences in diet composition or caloric intake is a subject of continued debate and has not been clearly delineated. In a highly controlled prospective cohort study, we set out to characterize phenotypic differences in maternal glucose and lipid metabolism between NW and OB pregnancies using tightly controlled gestational windows, 72 h of controlled eucaloric diets, and CGM metrics that have been associated with fetal overgrowth in diabetes and obesity [9]. Because NB%fat is viewed as a sensitive marker of intrauterine nutrient exposure [4] and better-predicts childhood obesity than BW [10], dual X-ray absorptiometry (DXA) was employed to measure NB%fat in infants and in women post-delivery. We tested the hypothesis that CGM metrics would reveal higher fasting and PP glucose (primary outcome) in women with OB vs. NW in *Early* (14–16 weeks) and *Later* (26–28 weeks) gestation despite controlled diets, and unique glucose metrics by CGM would add to prediction models of NB%fat that included maternal TG.

## 2. Materials and Methods

This was an NIH-funded prospective trial (R56 DK078645; R01 DK078645). Results from the primary analysis characterizing TG patterns during liquid-breakfast test meals between OB and NW groups and their role in predicting fetal fat accretion are reported elsewhere [8]. The analyses herein were planned a priori with the goal of ascertaining differences in CGM metrics on 3 days of a provided, macronutrient- and calorie-controlled diet and their prediction of NB%fat. The study was approved by the Colorado Multiple Institutional Review Board (COMIRB, #07-0535); all women gave their written informed consent. Persons who self-identified as women and were pregnant (18–35 years’) and their infants were studied at University of Colorado Hospital from 2009 to 2017. Fifty-four healthy English-speaking women with singleton pregnancies were enrolled (*n* = 27 NW; pre-pregnancy BMI 20–26 kg/m^2^ and *n* = 27 OB; pre-pregnancy BMI 30–38 kg/m^2^). To minimize the risk of growth restriction or preterm birth that would confound measures of infant body composition, all chronic medical conditions (i.e., hypertension, HIV, cardiac dysfunction) were exclusions. The women with OB were screened for glucose intolerance before enrollment [11] and were not included if they failed an early 100 g glucose tolerance test (OGTT). The term “women” is used in this manuscript because all of the participants identified themselves as such. The use of the term “women or maternal” in this manuscript is intended to be used inclusively to respect previously published reports.

Women were studied both *Early* (14–16 weeks) and *Later* (26–28 weeks) during pregnancy. They wore a CGM for 72 h while consuming a eucaloric diet matched for macronutrient composition. Three women with OB could not be studied *Later* due to gall bladder disease, pregnancy loss, or relocation. At 28 weeks, both groups underwent a 100 g OGTT to diagnose gestational diabetes (GDM) [11]; glucose and insulin were measured at baseline/fasting, 1, 2, and 3 h for insulin sensitivity estimates using assays previously reported [7,8]. Those who met the diagnostic criteria for GDM were excluded from this analysis. Only term (≥37 weeks), healthy NB were included in the final NB%fat analysis, given that fetal fat accretion was rapid near term. One NB from an NW mother was excluded due to pre-term delivery. Exclusions and birth complications were previously reported [8]; 26 NW and 19 OB offspring were included for term NB%fat analysis.

Macronutrient-controlled eucaloric diets: Ad libitum dietary fat and carbohydrate markedly affect glucose and lipids [12] and would be expected to confound the comparison of CGM metrics between groups. Thus, women were provided with standardized diets prepared by the Colorado Clinical Translational Science Institute (CCTSI) Bionutrition kitchen for 3 days while wearing a CGM. The 3-day diets for women with OB and NW were matched for calories and macronutrients: 50% carbohydrate (30% complex/20% simple carbohydrates; of total calories); 35% fat (12% saturated/12% monounsaturated/11% polyunsaturated); 15% protein. Energy requirements were based on the Institute of Medicine guidelines (OB: 25 kcal/kg; NW: 30 kcal/kg). During standard time periods, women consumed 25% of calories at breakfast (between 0600 and 1000), 30% at lunch (1100–1400), 35% at dinner (1700–2000), and 10% as a bedtime snack (after 2000). CGM metric analyses were completed within tight gestational windows of 14–16 and 26–28 weeks due to the progressive insulin resistance of pregnancy.

Other biochemical measures: The collection of fasting TG and PPTG data was previously reported [8] and were used in the regression analysis to determine if CGM metrics added predictive value to TGs in the relationship with NB%fat. Maternal insulin resistance (IR) from the OGTT was estimated using the product of glucose and insulin area-under-the-curve (AUC) [8] using the 28 week, 3 h, 100 g OGTT.

Blinded Continuous Glucose Monitoring: Interstitial glucose was initially measured using CGMS GOLD (Medtronic MiniMed, Symlar, CA, USA), followed by wireless iPro^®^1 and then iPro^®^2 (Medtronic MiniMed) when CGMS GOLD was no longer supported. Data procedures were applied with extraction of pregnancy-relevant glucose variables as previously described [13]. 24 h %time-in-range (%TIR) was defined as 63–140 mg/dL [14]. All CGM measures represent an average over 48–72 h during controlled diet. Despite precautions to avoid lost data, 3 NW participants did not have CGM data at 14 weeks, and 2 different participants did not have data at 28 weeks (*n* = 24 and *n* = 25 evaluable cases, respectively). In the OB cohort, there were *n* = 24 evaluable cases at 14 weeks (gallstones exclusion, 2 cases sensor malfunction) and *n* = 23 cases at 28 weeks (gallstones, intrauterine fetal demise, relocation, sensor malfunction).

Physical Activity: Physical activity was assessed at 14 and 28 weeks using the validated 36-item Pregnancy Physical Activity Questionnaire (PPAQ; One-week test–retest reliability demonstrated by intraclass correlations of 0.78–0.93) [15]. The women were asked not to vigorously exercise while CGMS was worn.

Delivery Outcomes and Maternal and Newborn Adiposity: Delivery outcomes including delivery type, complications, birth length, and BW were extracted from the medical record. Ponderal Index was calculated (weight [kg]/height^3^). Forty-five term NB underwent dual energy X-ray absorptiometry (DXA) at Children’s Hospital Colorado at ~2 weeks (mean 15.6 days, range = 12–20 days; QDR Discovery fan beam densitometer, Hologic Delphi-W, Hologic Inc., Waltham, MA, USA; Apex version 3.2 software), as described previously [8,16]. The DXA was performed at 2 weeks because of the expected newborn diuresis affecting total body water in the first week of life and the return of fat mass by 7 to 14 days [16,17]. In 2 NB, the DXA revealed a calibration error; for these measures, we applied a regression equation based on our previous data [16] to predict the NB%fat as previously described [8]. On the same day as the neonatal DXA, a maternal DXA was also performed (Hologic Delphi-W, Hologic Inc., Waltham, MA, USA).

Power Analysis: Power was calculated a priori (PASS 2005 software, Kaysville, UT, USA) to test the hypothesis that pregnancies affected by OB have higher fasting and postprandial glucose in *Early* and *Later* gestation. Based on our NIH-funded pilot study (R56 DK078645), 15 women/group would detect a between-group fasting-glucose difference of 6 ± 4.6 mg/dL (SD) *Early* and 14 ± 9.7 mg/dL *Later* for 84–91% power (α = 0.01) using a 2-sided/2-sample *t*-test. To detect a clinically meaningful 15 mg/dL difference in postprandial glucose in the NW vs. OB participants (α = 0.05, 1 − β = 0.8), 12 women per group were required.

Statistical Analyses: Area-under-the-curve (AUC) was calculated to represent total potential fetoplacental nutrient exposure [13] for CGM measures to characterize patterns of glycemia. Data are presented as mean ±SEM; between-group [OB minus NW] and within-group differences [*Later* minus *Early*] with 95% confidence intervals (CI) are provided for CGM and OGTT variables. All variables approximated a normal distribution with the exception of plasma insulin, which was log-transformed for analysis. Between-group differences were assessed using *t*-tests for independent groups and within-group differences by paired *t*-tests for primary and secondary outcomes. Correlations were assessed using Pearson’s *r.* For this analysis, multivariate regression models were constructed to include TG, maternal characteristics (maternal BMI at delivery, and %BF after delivery), insulin sensitivity derived from OGTT measures, and CGM metrics that demonstrated a between-group difference. Multiple and univariate linear regression were used to test for predictive associations (IBM SPSS Statistics v24, Armonk, NY, USA). For these analyses, *p* < 0.05 was considered statistically significant.

## 3. Results

Maternal and Newborn Characteristics: Fifty-four participants (27 per group) were studied during gestational week 15.9 ± 0.2 (*Early*) and 27.8 ± 0.1 (*Later*). Participants with NW and OB were similar in age, gravida, and were mostly Caucasian (Table 1). By design, participants with OB had a significantly higher BMI; however, gestational weight gain was similar between maternal BMI groups. There were no between-group differences in physical activity either *Early* or *Later* [8], as previously reported. The cohort with OB had a higher percentage of cesarean deliveries. All included infants were healthy and born at term (39.7 weeks, both groups). Infants born to mothers with OB had a significantly higher BW compared to those born to NW mothers (3528 g vs. 3258 g, *p* = 0.037, respectively). There was a higher percentage of females born to NW vs. OB (50% vs. 30%, *p* > 0.05). Although there was a trend for NB%fat at 2 weeks to be higher in neonates of those with OB vs. NW, the difference did not meet statistical significance (11.0 vs. 8.9%, *p* > 0.05). At 2 weeks postpartum, women with OB had significantly higher %fat compared to NW (41% vs. 33%, respectively, *p* < 0.0001).

**Figure 1:**
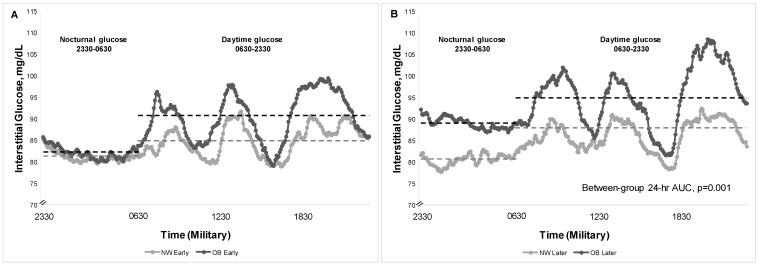
Patterns of 24 h glycemia measured by CGM in participants with NW and OB, both *Early* (14–16 weeks, Panel (**A**)) and *Later* (26–28 weeks, Panel (**B**)) in pregnancy. Gray and black dashed lines show mean nocturnal and daytime glucose between the groups.

Group Differences in Patterns of Glycemia by CGM: Table 2 and Figure 1 show differences in patterns of glycemia between maternal groups *Early* and *Later* in gestation. On the eucaloric diets, there were no between-group differences in fasting glucose by CGM *Early* or *Later* in gestation. Our hypothesis was largely supported by a pattern of higher PP glucose responses in women with OB both *Early* and *Later*. This was true across individual meals and as an average across 1 h and 2 h PP glucose. In Later gestation, women with OB averaged a 10 mg/dL statistically higher 1 h and 2 h PP glucose across meals (Table 2) compared to NW.

In *Early* gestation, women with OB had a higher daytime mean glucose (91.0 vs. 84.8 mg/dL [95% CI for difference: 1.10, 10.53]) vs. NW and a correspondingly higher daytime glucose AUC (Table 2, *p* = 0.01 for both). This was explained by a pattern of higher 1 h PP (100.2 vs. 93.4 mg/dL [95% CI: −0.06, 13.66]) and 2 h PP glucose (95.3 vs. 87.3 mg/dL [95% CI: 2.65, 13.42,) in OB vs. NW, respectively, because nocturnal AUC was similar (Figure 1). The % TIR (63–140 mg/dL) was similar in women with OB (97.7 ± 1.1%) compared to those with NW (98.5 ± 1.1%)(95% CI: −3.21, 1.60).

In *Later* gestation, women with OB averaged a 10 mg/dL statistically higher 1 h PP glucose across meals (109.0 mg/dL vs. 98.3 mg/dL [95% CI for difference:, 4.0, 16.94]) as well as 2 h PP glucose (101.2 vs. 92.0 mg/dL [95% CI: 3.48, 15.40] compared to NW, but much lower than current gestational diabetes treatment targets [14,18]. By *Later* gestation, women with OB had higher glycemia across the 24 h period with a higher 24 h mean glucose (93.0 vs. 85.4 mg/dL, respectively [95% CI: 3.52, 13.14]), and a correspondingly higher 24 h glucose AUC by 9% (*p* = 0.001 for both, Table 2, Figure 1). The OB group vs. NW *Later* also had higher mean nocturnal glucose (88.5 vs. 81.3 [95% CI: 1.43, 12.84]; *p* = 0.015). Importantly, the %TIR remained similar between groups (OB: 98.8 ± 0.5%; NW: 95.1 ± 1.7% [95% CI: −0.03, 7.32]).

Group Differences in Metabolic Measures: As previously reported, the fasting, 1 h, and 2 h PP TG were ~30% different between groups both *Early* and *Later* [8]. In response to the 3 h 100 g OGTT, women with OB had significantly higher fasting plasma glucose and (log)insulin compared to NW (83.0 vs. 77.3 mg/dL, respectively [95% CI for difference: 1.89, 9.20]), followed by ~20 mg/dL higher 1 and 2 h post-load glucose concentrations (*p* = 0.004–0.01, Table 1). Although the post-load insulin concentrations appeared higher in OB vs. NW, the log-transformed concentration differences were not statistically significantly different. Participants with OB were more insulin-resistant compared to those with NW, demonstrated by higher 3 h glucose AUC and a higher glucose × insulin AUC product (Table 1).

Within-group Changes in Glycemia from Early to Later Gestation: Table 2 shows within-group changes in patterns of glycemia for the NW and OB groups. Notably, fasting glucose did not change in NW from *Early* to *Later* (81.3 to 81.0 mg/dL), and the slight increase in fasting glucose in the OB group *Early* to *Later* (82.7 to 86.0 mg/dL) was not statistically significant. In NW, the 1 h PP glucose across all meals did not increase statistically from *Early* to *Later* (93.4 vs. 98.3 mg/dL) but did statistically increase in the OB group (100.1 to 109.0 mg/dL, *p* = 0.027). The peak PP breakfast excursion (highest PP glucose within 2 h [13]) increased statistically from *Early* to *Later* within the NW group (8.8 to 15.0 mg/dL, *p* = 0.047) without a change in fasting glucose. In the OB group, the peak PP breakfast excursion increased from *Early* to *Later* (11.1 to 24.0 mg/dL, *p* = 0.024), but the maximum peak PP excursion in either group, at any time point, or after any meal was only 26 mg/dL. The pregnancies affected by OB demonstrated a pattern of increased glycemia postprandially over time between *Early* and *Later* gestation, particularly after breakfast, in 1 h PP measures, mean nocturnal and 24 h glucose, and in nocturnal and 24 h AUC glucose (all *p* < 0.05; Table 2).

### Correlates with Neonatal Birthweight and Adiposity

Total Cohort: Across 45 mother–infant pairs, none of the CGM or metabolic measures were correlated with infant BW. In addition, maternal characteristics, including pre-pregnancy BMI and gestational weight gain (GWG), were not correlated with infant BW or NB%fat. Across women in *Later* pregnancy, there was a pattern of moderate correlation between PP meal responses and NB%BF (1 h PP dinner [r = 0.331, *p* = 0.02], 2 h PP breakfast [r = 0.322, *p* = 0.03], 2 h PP dinner AUC [r = 0.331, *p* = 0.02]. Moreover, the 24 h glucose AUC (r = 0.310, *p* = 0.04) and mean 24 h glucose (r = 0.305, *p* = 0.04) were modestly correlated with NB%fat.

By Group: There were no correlations between any CGM measures and NB%fat in those with NW *Early*. In *Later* pregnancy, the PP breakfast excursion (~15 mg/dL; Table 2) was correlated with NB%fat (r = 0.519, *p* = 0.008) in NW. In the OB group, the *Early* 2 h PP glucose across meals was correlated with NB%fat (r = 0.469, *p* = 0.05 [borderline significant]). By *Later* gestation, significant correlations between the mean 24 h glucose and NB%fat (r = 0.538, *p* = 0.02), and 24 h glucose AUC and NB%fat (r = 0.532, *p* = 0.02) were demonstrated in the OB group.

Predictors of Neonatal Adiposity by Univariate and Multivariate Regression: There were no correlations between insulin sensitivity estimates based on the 100 g OGTT and NB%fat, nor were there correlations between GWG or maternal %BF after delivery or with NB%fat. As reported previously [8], across the 45 mother–infant pairs, a 1 h or 2 h PPTG both *Early* and *Later* in gestation predicted NB%fat and explained ~30% of the variance, respectively (R^2^ = 0.32 and R^2^ = 0.29, *p* < 0.001 for both) [8]. In the OB cohort, the *Early* 1 h or 2 h PPTG predicted 50% of the variance in NB%fat (R^2^ = 0.50; *p* < 0.01) [8]. None of the CGM or glucose variables here added predictive value to the fasting and PPTG measures on NB%fat across the cohort or in mothers with OB specifically.

## 4. Discussion

We set out to evaluate the premise that pregnancies affected by OB have higher patterns of glycemia using CGM metrics associated with fetal overgrowth [9], even when diets are carefully controlled. In this parallel-group comparative study of OB and NW pregnant women without significant co-morbidities, the gestational week of measurement was fixed at 14–16 and 26–28 weeks to reduce variability from the expected weekly increase in insulin resistance. Notably, a controlled diet that was both eucaloric and equivalent in macronutrient composition was provided for 3 days to further reduce variation in patterns of glycemia from ad libitum diet consumption that would be expected to influence CGM metrics. Both *Early* and *Later* in gestation, pregnancies with OB manifested higher glycemia despite highly controlled diets compared to their NW counterparts, supporting our a priori hypothesis. *Early* in pregnancy, fasting and nocturnal glucose was similar between groups but participants with OB had higher PP meal responses. Between *Early* and *Later* pregnancy, both groups of women demonstrated higher nocturnal glucose and increased PP glucose responses. Those with OB had larger increases, such that by 26–28 weeks gestation, CGM metrics demonstrated glucoses that were consistently statistically higher after meals, over the 24 h period, and throughout the night. Remarkably, the 1 h PP (109.0 vs. 98.3 mg/dL) and the 2 h PP response (101.2 vs. 92.0 mg/dL) averaged across all meals *Later* in both the OB and NW group, respectively, were much lower than current 1 h PP and 2 h PP therapeutic targets for diabetes in pregnancy (<140 mg/dL, <120 mg/dL, respectively [18]), and were similar to our previously reported normative data [6]. CGM predictors of NB%fat measured by DXA were identified: In women with NW, only the *Later* gestation PP breakfast response (~15 mg/dL) was positively correlated with NB%fat. The strongest correlation in OB *Later* was the 24 h mean glucose and the 24 h glucose AUC.

Because the majority of large-for-gestational age (LGA) deliveries are accounted for by pregnancies with OB rather than GDM [3], we sought to examine differences in CGM metrics with a macronutrient- and calorie-controlled diet. This otherwise healthy cohort with OB displayed normal glucose tolerance [18] and demonstrated lower patterns of glycemia than expected, likely due to consuming the provided eucaloric diet. Fasting glucose by CGM was slightly higher in the OB group *Later*, but did not reach statistical significance. However, fasting glucose on the OGTT at 28 weeks was higher in the OB group as was the mean nocturnal glucose *Later* in pregnancy. Moreover, on the controlled diet, the PP meal excursions in both groups *Early* in gestation were surprisingly low (9–16 mg/dL for NW, 11–19 mg/dL for OB). Similarly, the 1 and 2 h PP glucoses across meals in the NW group throughout pregnancy was ~30–40 mg/dL below current targets, and in the OB group, they were ~20–30 mg/dL below targets. In our previously published systematic review [6], the pattern of glycemia in late normal pregnancy (~34 weeks, BMI range 22–28 kg/m^2^) was lower than had been formerly appreciated: fasting glucose was 71 ± 8, 1 h PP glucose was 109 ± 13, 2 h PP glucose was 99 ± 10, and mean 24 h glucose was 88 ± 10 mg/dL (mean ± SD). In this current study, while on a eucaloric diet, both groups of women fell within these ranges. In another of our previous studies, women with NW (*n* = 22) and OB (*n* = 16) wore a CGM while consuming a controlled diet and in addition, while consuming their typical ad libitum diet. Analysis of the CGM data was limited to only 1 day of each diet. In that observational study, those with OB (vs. NW) had higher fasting glucose both *Early* and *Later*, and higher 24 h patterns of glycemia by ~9% on ad libitum and ~8% when diet was controlled [7]. In this current study, 72 h of CGM data on controlled study diets produced patterns of 24 h glycemia that were 13–14% lower compared to the previous study [7]. Taken together, these data suggest that lower glucose concentrations may be achieved by consuming a healthy eucaloric diet pattern for a longer period (3 days) across pregnancy in both NW and OB individuals, effectively blunting peak PP excursions in the range of 9–26 mg/dL.

Others have employed CGM technology in pregnancy to characterize 24 h glucose patterns, but this is the only study to have provided controlled diets to participants with NW and OB, both *Early* and *Later* in pregnancy, within tightly controlled gestational windows. Chandler-Laney and colleagues [19] studied 40 pregnant Black women (BMI 21.3–43.9 kg/m^2^, 32.0–34.6 weeks) who wore a CGM while consuming an ad libitum diet; the fasting glucose was similar to women with OB in this study (86.5 ± 12.7 mg/dL, mean ± SD), but glycemia in NW vs. OB was not reported. In a randomized cross-over study, Kizirian and colleagues [20] studied 17 women in Australia with risk factors for GDM (BMI 23.8 ± 4.7 kg/m^2^, 29.3 ± 1.3 weeks) who were provided a low glycemic- and a higher-glycemic-load diet while wearing a CGM for 24 h each. The %TIR (70–140 mg/dL) was 95.1 ± 1.7% on the low-glycemic-load diet day (vs. 87.7 ± 3.2% on higher-glycemic-load day, *p* = 0.031), and the low-glycemic-load diet %TIR was similar to both groups of women, both *Early* and *Later*, in this study. This high %TIR also coincides with a recent report in uncomplicated pregnancies [21]. While our controlled diets were not designed based on glycemic load, the diets contained 50% of total energy from carbohydrates, the majority being complex carbohydrates (30% of total calories) with mostly low–medium-glycemic index foods.

Maitland and colleagues [22], in the UK, conducted a 3-arm randomized trial (*n* = 16; BMI 37 ± 4.7 kg/m^2^, 24–28 weeks) in women with OB, in which one arm involved consuming an ad libitum diet (2 days) while wearing a CGM. Women in that study demonstrated lower glucose than women with OB in this study (fasting glucose 76, 24 h mean glucose 85, daytime mean 87, and nocturnal mean 78 mg/dL). In Brazil, Rahmi and colleagues [23] studied 10 women with NW (pre-gestational BMI 22.1 [range 21.7–23.8] kg/m^2^) and 10 women with OB (39.9 [35.8–41.9]) who wore a CGM for 3 days while on an ad libitum diet at ~25 weeks gestation (range 24–28 weeks). Although diet was not controlled, their findings mirror those in this study, wherein women with OB showed higher glycemia over 24 h compared to those with NW. While the women with NW [23] demonstrated similar glucose patterns to the NW women in our study, the women with OB demonstrated lower PP glucose (by ~9 mg/dL) across 24 h compared to OB in our study. Finally, in the recently reported large observational prospective cohort by Durnwald and colleagues [24], pregnant individuals wore GCM throughout gestation, and glycemic metrics were compared in the individuals who developed GDM versus those who did not. Although NW vs. OB pregnancies were not compared, the %TIR in the individuals who did not develop GDM was 93–94% in the first and third trimester of pregnancy, slightly less than our range in both NW and OB groups (95–99%). However, mean glucose was higher in the first and third trimesters in the individuals who did not develop GDM (101 and 99 mg/dL, respectively) compared to women in this study with NW and OB at 14–16 weeks (83 and 88 mg/dL, respectively) and at 26–28 weeks (85 and 93 mg/dL, respectively) [24]. This difference might also be related to the eucaloric diet consumed by our participants as opposed to the ad libitum diet. None of the above studies measured NB%fat.

Newborn adiposity is a marker sensitive to intrauterine nutritional exposures and more strongly predicts risk for childhood obesity than BW [4,10]. Unique to this study was our measurement of NB%fat by DXA, which some experts still consider the gold standard in precision for infant body composition [16], to evaluate associations between CGM glucose metrics and fetal growth. While BW was higher in the offspring of women with OB (vs. NW), the difference in NB%fat did not reach statistical significance (11.0% vs. 8.9%). Nonetheless, when the NB%fat was used as a continuous dependent variable, some CGM predictors were revealed in this NW and OB population with relatively low glycemic patterns. Across the total cohort, PP glucose and 24 h glycemia at 26–28 weeks gestation were correlated with NB%fat. Unique to women with NW, the PP breakfast excursion during 26–28 weeks was associated with NB%fat. In women with OB, the average 2 h PP glucose response across meals (95 mg/dL) was already associated with NB%fat at 14–16 weeks gestation. In those with OB, at 26–28 weeks, the average 24 h glucose and 24 h glucose AUC explained ~28% of the variance in NB%fat (r = 0.538, r = 0.532, respectively). However, linear regression models constructed using the fasting TG and PPTG data from our previously reported breakfast test meal studies [8] demonstrated that the 1 h and 2 h PP TG at 14–16 weeks gestation were the strongest predictors of NB%fat. Adding insulin sensitivity estimates from the 100 g OGTT and CGM metrics from this study did not improve the predictive model. Given that both glucose and TG are sensitive to maternal diet patterns, and both increase postprandially from the ingestion of simple sugars, these data add evidence to suggest that diet intervention across pregnant women, and particularly in women with OB, might be more intentionally targeted throughout pregnancy to mitigate fetal overgrowth patterns.

This study has strengths and limitations. Incorporating measures of fasting TG and PPTG and estimates of insulin resistance to the CGM metrics to predict fetal overgrowth are strengths, as well as utilizing DXA to measure NB%fat. Although BW was higher in the offspring of women with OB, we did not find a between-group difference in %NBfat. This may be in part due to a higher percentage of female offspring being born to NW mothers (50%) compared to OB mothers (30%) and females tend to have higher NB%fat compared to males. We did not have adequate sample size to evaluate sex differences. Furthermore, the measurement of NB%fat was at ~2 weeks of life, which might have been influenced by early feeding patterns and partially account for the lack of difference in adiposity. Providing a highly controlled maternal diet while wearing the CGM allowed us to minimize the confounding variable calories and macronutrients which substantially influence CGM metrics between the NW and OB groups but could also limit generalizability to ad libitum diet conditions. CGM technology has evolved since this study was conducted with more precise sensors, but focusing on between-group comparisons rather than attempting to define precise individual time point measures would seem to attenuate this limitation.

## 5. Conclusions

On eucaloric diets matched for macronutrient composition, pregnancies affected by OB demonstrated higher patterns of 24 h glycemia both *Early* and *Later* in pregnancy. While PP glucose responses increased within NW women from *Early* to *Later*, the patterns across 24 h increased with greater magnitude within OB, supporting our hypothesis that the OB metabolic phenotype contributes to higher 24 h glycemia in pregnancy, independent of dietary macronutrient composition and calories. However, despite these differences, mean glucose and PP excursions in both groups were saliently lower than current therapeutic targets for diabetes in pregnancy when macronutrients and calories were controlled. *Later* in pregnancy, the mean and 24 h glucose AUC correlated with NB%fat in OB. However, the addition of CGM metrics in this study did not contribute to the prediction of NB% fat beyond fasting and PPTG measures in NW and OB pregnancies. This observation supports the premise that lipid metabolism may be at least as, if not more, important than glucose metabolism in predicting fetal overgrowth in OB pregnant populations without diabetes. Given both groups exhibited high %TIR (95–99%) both *Early* and *Later* in pregnancy when defined as 63–140 mg/dL, a lower %TIR range may be necessary to differentiate glycemic patterns associated with fetal overgrowth in NW vs. OB individuals without pre-existing diabetes for GDM. Because early nutritional interventions that extend through delivery which limit simple carbohydrate and saturated fats are likely to have favorable effects on both TG and glucose patterns, nutritional interventions may be important not only in GDM, but also in pregnancies affected by OB, at high risk for fetal overgrowth and offspring metabolic disease.

## Figures and Tables

**Table 1:** Maternal biochemical characteristics during *Early* (14–16 weeks) and *Later* (26–28 weeks) gestation, delivery, and postpartum. *p*-values are for between-group comparisons at the same timepoint (NS = *p* > 0.05). Data are mean ± SEM. For OGTT variables, between-group [OB minus NW] and within-group differences [*Later* minus *Early*] with 95% confidence intervals (CI) are provided.

	NW (*n* = 27)		Obese (*n* = 27)		NW vs. OB, Same Time Point *p*-Value Difference [95% CI]
Baseline Maternal Characteristics					
Age (years)	31 ± 0.6		30 ± 0.8		NS
Pre-pregnancy BMI (kg/m^2^)	22.3 ± 0.3		32.0 ± 0.6		<0.0001
Primigravida (%total)	52		41		NS
Caucasian (%total)	93		93		NS
*Early* and *Later* Maternal Measures	*Early*	*Later*	*Early*	*Later*	
Fasting TG, mg/dL *	89.2 ± 3.98	135.1 ± 7.8	126.2 ± 8.7 ^†^	174.9 ± 12.2 ^‡^	-
1 h PP TG, mg/dL *	95.3 ± 4.6	153.2 ± 8.0	143.4 ± 10.8 ^†^	201.2 ± 13.3 ^‡^	-
2 h PP TG, mg/dL *	86.6 ± 5.2	137.9 ± 8.1	135.3 ± 10.7 ^†^	189.1 ± 13.1 ^†^	-
100 g OGTT, 28 weeks					
Fasting glucose, mg/dL		77.3 ± 1.2		83.0 ± 1.4	0.004 5.54 [1.89, 9.20]
1 h glucose		121.0 ± 5.7		144.0 ± 3.9	0.002 22.85 [8.74, 36.96]
2 h glucose		104.0 ± 3.5		120.1 ± 5.0	0.01 16.24 [4.14, 28.35]
3 h glucose		90.0 ± 3.9		98.0 ± 5.1	NS 8.09 [−4.83, 21.00]
3 h glucose AUC, mg × dL/h		18,616 ± 536		21,243 ± 540	0.001 2626 [1095, 4157]
Fasting insulin, uIU/L		11.1 ± 1.0		19.8 ± 2.0	0.01 8.60 [4.33, 12.87]
1 h insulin		98.0 ± 18.0		136.4 ± 13.4	NS 38.75 [−6.58, 84.08]
2 h insulin		80.0 ± 7.5		118.4 ± 18.1	NS 38.80 [0.40, 77.19]
3 h insulin		46.0 ± 6.6		76.4 ± 11.2	NS 30.42 [4.71, 56.12]
3 h insulin AUC, uIU × h/L		12,479 ± 1471		18,173 ± 1891	NS 5694 [918, 10,470]
GlucoseAUC × InsulinAUC		248,118,444 ± 33,167,536		396,303,263 ± 47,676,620	0.013 148,184,819 [32,145,562, 264,224,075]
Delivery and Postpartum					
Gestational weight gain, kg	13.7 ± 0.8		14.2 ± 1.6		NS
Gestational Age at Delivery, weeks	39.7 ± 0.2		39.7 ± 0.2		NS
Cesarean (%total)	23		40		NS
Birthweight, g	3258 ± 74 (*n* = 26)		3528 ± 107 (*n* = 20)		0.037
Female (% total)	50		30		NS
Ponderal Index	2.6 ± 0.04		2.7 ± 0.05		0.03
2 weeks NB %fat	8.9 ± 0.7		11.0 ± 1.2 (*n* = 19)		NS
2 weeks Total mass, g	3864.8 ± 95.4		4123 ± 137		NS
2 weeks Maternal BMI, kg/m^2^	24 ± 0.4		33 ± 0.5		<0.0001
2 weeks Maternal %fat	33 ± 1		41 ± 1		<0.0001

95% CI = 95% confidence interval; NS = *p* > 0.05. * After 3 days of control diet while wearing a blinded CGM at 14–16 and 26–28 weeks, women reported to the CCTSI clinic after fasting × 10 h. Baseline labs were collected, followed by a liquid breakfast shake (30% total calories) and frequent blood sampling over 4 h for plasma TG using assays previously reported [7,8] in the CCTSI Laboratory (paired samples run on same batch). ^†^ *p* ≤ 0.001, NW vs. OB same timepoint. ^‡^ *p* < 0.01, NW vs. OB same timepoint.

**Table 2:** Maternal CGM metrics from *Early* (14–16 weeks) and *Later* (26–28 weeks) gestation. Data are mean ± SEM. Between-group [OB minus NW] and within-group differences [*Later* minus *Early*] with 95% confidence intervals (CI) are provided.

	NW *Early*	OB *Early*	NW vs. OB *Early* *p*-Value Difference [95% CI]	NW Later	OB Later	NW vs. OB *Later* *p*-Value Difference [95% CI]	NW *Early* vs. NW *Later* * p-Value Difference [95% CI]	OB *Early* vs. OB *Later* * p-Value Difference [95% CI]
*n*	24	24		25	23			
Fasting glucose ^†^	81.3 ± 1.9	82.7 ± 2.1	1.47 [−4.20, 7.14]	81.0 ± 1.9	86.0 ± 2.3	4.8 [−1.27, 10.80]	−0.41 [−4.55, 3.74]	2.45 [−0.89, 3.27]
Meals								
Preprandial Lunch	78.3 ± 1.7	85.0 ± 3.7	6.78 [−1.23, 14.78]	80.1 ± 1.9	84.0 ± 1.8	3.7 [−1.67, 9.10]	2.1 [−2.76, 6.91]	−2.06 [−10.72, 6.61]
Preprandial Dinner	78.0 ± 1.7	82.9 ± 3.0	4.93 [−2.04, 11.91]	77.2 ± 1.2	82.4 ± 1.7	0.016 5.21 [1.01, 9.40]	−1.66 [−4.98, 1.65]	−1.55 [−9.41, 6.30]
1 h PP breakfast	90.3 ± 2.8	99.0 ± 2.9	0.036 8.59 [0.60, 16.58]	96.0 ± 2.1	112.2 ± 4.1	0.001 16.2 [7.21, 25.25]	0.033 5.37 [0.46, 10.28]	0.010 12.6 [3.31, 21.90]
1 h PP lunch	96.0 ± 2.9	100.5 ± 3.3	4.59 [−4.24, 13.41]	100.0 ± 2.2	106.1 ± 2.4	0.049 6.57 [0.04, 13.10]	3.55 [−3.21, 10.3]	5.20 [−3.54, 13.94]
1 h PP dinner	94.0 ± 2.9	101.6 ± 2.3	0.042 7.8 [0.31, 15.30]	99.6 ± 2.2	108.1 ± 3.4	0.038 8.53 [0.49, 16.58]	4.04 [−3.45, 11.52]	5.89 [−1.98, 13.76]
2 h PP breakfast	85.2 ± 2.3	90.4 ± 2.3	5.24 [−1.23, 11.72]	89.2 ± 2.1	100.2 ± 3.1	0.005 11.0 [3.51, 18.47]	3.99 [−1.75, 9.73]	0.02 7.21 [1.29, 13.14]
2 h PP lunch	89.1 ± 2.2	94.6 ± 2.3	5.47 [−0.94, 11.89]	92.6 ± 2.0	98.1 ± 2.4	5.55 [−0.69, 11.78]	2.99 [−2.22, 8.21]	3.74 [−3.10, 10.58]
2 h PP dinner	87.6 ± 2.1	101.0 ± 2.8	<0.0001 13.24 [6.25, 20.23]	93.5 ± 2.1	105.2 ± 3.2	0.003 11.78 [4.23, 19.33]	0.036 5.39 [0.40–10.39]	4.67 [−3.42, 12.77]
1 h PP across 3 meals	93.4 ± 2.6	100.2 ± 2.2	0.052 6.80 [−0.06, 13.66]	98.3 ± 1.8	109.0 ± 2.7	0.002 10.44 [4.0, 16.94]	4.32 [−1.19, 9.82]	0.027 8.02 [1.02, 15.01]
2 h PP across 3 meals	87.3 ± 1.8	95.3 ± 2.0	0.004 8.04 [2.65, 13.42]	92.0 ± 1.6	101.2 ± 2.5	0.003 9.44 [3.48, 15.40]	0.039 4.13 [0.22, 8.03]	5.08 [−0.74, 10.90]
PP excursion breakfast	8.8 ± 2.7	11.1 ± 3.0	2.35 [−5.81, 10.52]	15.0 ± 2.2	23.5 ± 4.3	9.01 [−0.42, 18.46]	0.047 4.83 [0.07, 9.59]	0.024 12.88 [1.84, 23.92]
PP excursion lunch	17.8 ± 3.1	15.5 ± 3.9	−2.28 [−12.27, 7.71]	19.4 ± 1.5	22.3 ± 1.9	2.86 [−1.94, 7.66]	1.38 [−6.21, 9.0]	7.26 [−2.80, 17.32]
PP excursion dinner	15.9 ± 2.6	18.8 ± 2.9	2.87 [−4.97, 10.72]	22.3 ± 1.9	26.0 ± 2.5	3.34 [−2.88, 9.57]	5.70 [−1.60, 12.99]	7.46 [−1.95, 19.87]
Diurnal								
Daytime Mean glucose	84.8 ± 1.6	91.0 ± 1.8	0.017 5.82 [1.10, 10.53]	86.9 ± 1.5	94.5 ± 1.7	0.002 7.60 [2.96, 12.24]	1.24 [−1.84, 4.34]	3.09 [−1.02, 7.19]
Nocturnal glucose	81.6 ± 1.5	83.1 ± 1.8	1.53 [−3.20, 6.26]	81.3 ± 2.2	88.5 ± 1.7	0.015 7.13 [1.43, 12.84]	0.07 [−3.36, 3.50]	0.020 4.63 [0.80, 8.47]
Mean 24 h glucose	83.8 ± 1.4	88.0 ± 1.7	4.21 [−0.25, 8.67]	84.5 ± 1.7	93.0 ± 1.7	0.001 8.33 [3.52, 13.14]	0.43 [−2.53, 3.41]	0.03 4.19 [0.44, 7.94]
Time in range, 63–140, % of 24 h	98.5 ± 1.1	97.7 ± 1.1	−0.81 [−3.21, 1.60]	95.1 ± 1.7	98.8 ± 0.5	0.052 3.65 [−0.03, 7.32]	0.023 −4.45 [−8.21, −0.68]	0.50 [−1.99, 2.99]
AUCs								
2 h AUC breakfast	10,737 ± 288	11,485 ± 269	748 [−44.62, 1541]	11,048 ± 213	12,572 ± 347	<0.0001 1523 [719, 2327]	278 [−220, 775]	0.017 944 [190, 1698]
2 h AUC lunch	10,955 ± 241	11,565 ± 307	610 [−173, 1392]	11,160 ± 239	11,859 ± 260	0.054 699 [−11.0, 1409]	177 [−383, 738]	342 [−501, 1186]
2 h AUC dinner	10,792 ± 284	11,763 ± 273	0.018 971 [178, 1763]	11,167 ± 190	12,118 ± 362	0.022 951 [146, 1756]	233 [−409, 874]	306 [−510, 1122]
24 h AUC	120,622 ± 1968	126,297 ± 2451	5675 [−611, 11,961]	120,993 ± 2386	133,320 ± 2364	0.001 12,328 [5558, 19,098]	275 [−3637, 4187]	0.029 6020 [700, 11,339]
Daytime AUC	86,341 ± 1564	92,109 ± 1783	0.019 5768 [1007, 10,530]	87,961 ± 1523	95,988 ± 1762	0.001 8027 [3359, 12,694]	1216 [−1797, 4228]	3238 [−969, 7445]
Nocturnal AUC	33,683 ± 758	34,543 ± 741	859 [−1274, 2993]	33,781 ± 912	36,739 ± 724	0.016 2958 [587, 5329]	269 [−1246, 1783]	0.021 1906 [318, 3495]

* Within-group (*Early* to *Later*) difference, paired *t*-tests, (19–22 cases). 95% CI = 95% confidence interval. ^†^ Fasting glucose was defined as the average six consecutive values starting at 06:00 h and/or after at least 7 h fasting. All CGM metric definitions have been previously published [13]. *p*-values ≤ 0.05 are reported.

## Data Availability

Some or all datasets generated during and/or analyzed during the current study are not publicly available but are available from the corresponding author on reasonable request. The data are not publicly available due to ongoing pre-planned analyses that are not yet complete.
